# Genome assembly and annotation of the European earwig *Forficula auricularia* (subspecies B)

**DOI:** 10.1093/g3journal/jkac199

**Published:** 2022-08-16

**Authors:** Upendra R Bhattarai, Mandira Katuwal, Robert Poulin, Neil J Gemmell, Eddy Dowle

**Affiliations:** Department of Anatomy, University of Otago, Dunedin 9016, New Zealand; Department of Anatomy, University of Otago, Dunedin 9016, New Zealand; Department of Zoology, University of Otago, Dunedin 9016, New Zealand; Department of Anatomy, University of Otago, Dunedin 9016, New Zealand; Department of Anatomy, University of Otago, Dunedin 9016, New Zealand

**Keywords:** *Forficula auricularia*, hybrid genome assembly, repeatome, genome annotation

## Abstract

The European earwig *Forficula auricularia* is an important model for studies of maternal care, sexual selection, sociality, and host–parasite interactions. However, detailed genetic investigations of this species are hindered by a lack of genomic resources. Here, we present a high-quality hybrid genome assembly for *Forficula auricularia* using Nanopore long-reads and 10× linked-reads. The final assembly is 1.06 Gb in length with 31.03% GC content. It consists of 919 scaffolds with an N50 of 12.55 Mb. Half of the genome is present in only 20 scaffolds. Benchmarking Universal Single-Copy Orthologs scores are ∼90% from 3 sets of single-copy orthologs (eukaryotic, insect, and arthropod). The total repeat elements in the genome are 64.62%. The MAKER2 pipeline annotated 12,876 protein-coding genes and 21,031 mRNAs. Phylogenetic analysis revealed the assembled genome as that of species B, one of the 2 known genetic subspecies of *Forficula auricularia*. The genome assembly, annotation, and associated resources will be of high value to a large and diverse group of researchers working on dermapterans.

## Introduction

Insects have been at the forefront of genetic research for various biological questions (Wilson-Sanders 2011; Mukherjee *et al.* 2015; Simons and Tibbetts 2019). However, most of the genetic studies are carried out on a small number of holometabolous insects that undergo true metamorphosis. In contrast to Holometabola, hemimetabolous insects undergo incomplete metamorphosis with a series of nymphal molts that increasingly resemble the adult form (Truman 2019). It is widely accepted that Holometabola branched out from hemimetabolous ancestors during the Permian 300 Mya (Labandeira and Phillips 1996; Yang 2001). Yet the conserved mode of development, embryonic organization, and the adult body plan of hemimetabolous insects offer a unique model for the study of developmental and evolutionary mechanisms. However, even with the increasing number of sequenced genomes, the majority belong to the Holometabola (Ylla *et al.* 2021). This has been a bottleneck for the exploration of the diverse biology and life history of hemimetabolous insects. To address this paucity, we report a high-quality annotated genome of the European earwig, *Forficula auricularia* (Dermaptera: Forficulidae).

The European earwig *F. auricularia* is widely distributed, comprising 2 recognized subspecies, A and B (Wirth *et al.* 1998). They are native to the western Eurasian region and were introduced to North America, Australia, and New Zealand, where they have quickly adapted and became abundant throughout the regions (Quarrell *et al.* 2018; Tourneur and Meunier 2019). The 2 subspecies A and B differ through mitochondrial divergence and in their reproductive life histories (Guillet, Guiller, *et al.* 2000; Guillet, Josselin, *et al.* 2000). Subspecies A is found in relatively colder climates and is univoltine with a long gregarious phase, whereas subspecies B is found in temperate and oceanic climates and is bivoltine (Lamb and Wellington 1974). In laboratory conditions, they fail to produce offspring by cross mating (Wirth *et al.* 1998). Their propensity to dwell on flower and kitchen gardens can cause significant damage to crops, flowers, and commercial vegetables and make them important agricultural pests (Campos *et al.* 2011; Hill *et al.* 2019).

They have been of particular interest for many researchers not just because of their importance in the agricultural ecosystem (Binns *et al.* 2021) but also their importance as a research model for various biological and evolutionary phenomena like sexual selection, maternal care, family interactions, reproductive strategy, and social behavior (Forslund 2000; Falk *et al.* 2014; Kramer *et al.* 2015; Van Meyel and Meunier 2020). They have been extensively studied by behavioral ecologists for the early evolution of group-living and family life (Falk *et al.* 2014). The male earwigs also show an unusual bias in their use of lateral left and right sexual organs without any conspicuous anatomical differentiation (Kamimura 2006). Like the right-handedness in humans, 90% of males of giant earwig *Labidura riparia* show a preference for the right penis for copulation, providing insights into the evolutionary origin of lateralization (Kamimura *et al.* 2021). Similarly, they are an excellent lab model to study extended phenotypes as they exhibit strange suicidal water-seeking behavior during the late stages of infection by mermithid nematodes (Herbison *et al.* 2019). However, their use as a genetic model has been severely limited by the lack of a reference genome.

Here, we have sequenced, assembled, annotated, and analyzed the genome of the European Earwig, *F. auricularia* and confirmed the subspecies identity of the individuals we used. This genome will help researchers study multiple facets of this insect’s exciting biology and evolutionary characters and broaden our understanding of insect and genome evolution.

## Methods and materials

### Sample collection and preparation

Earwigs (*F. auricularia*) were field collected from the Dunedin Botanic Garden (−45°51′27.59″S, 170°31′15.56″E) and reared in a temperature-controlled room (temperature: cycling from 15 to 12°C, day/night; photoperiod of L:D 16:8) in the Department of Zoology, University of Otago, Dunedin. Earwigs were snap-frozen in liquid nitrogen and stored at −80°C before dissection and subsequent nucleotide extraction. Earwigs were dissected in 1× PBS buffer under a dissection microscope to check for nematode parasites, and only nonparasitized individuals were used in this study. The head, wings and muscles from the thorax region were used for DNA extraction to avoid the gut microbiota. Juvenile instars required for RNA extraction were obtained directly from the field.

### DNA extractions

DNA was extracted using either the Nanobind Tissue Big DNA kit (Circulomics, USA) for high molecular weight DNA or DNeasy Blood & Tissue Kit (Qiagen, Germany) by following the manufacturer’s protocol. Tissues from a single individual were used for each extraction. After the extraction, RNase treatment was performed using 4 µl of RNase A (10 mg/ml) per 200 µl of DNA elute. DNA was quantified in a Qubit 2.0 Fluorometer (Life Technologies, USA) and quality analyzed using Nanodrop. Low-quality DNA samples were further cleaned with 1.8× by volume AMPure XP beads (Beckman Coulter, USA), wherever applicable, following the manufacturer’s instructions and eluted in 55 µl of molecular grade water. High-quality DNA samples were stored at −20°C and were used within a week of extraction.

### Linked-read library preparation and sequencing

Linked-read library was prepared at the Genetic Analysis Services (GAS), University of Otago (Dunedin, New Zealand). DNA from an adult male was extracted using the Nanobind kit and size-selected for fragments over 40 kbp using Blue Pippin (Sage Science, USA). A 10× linked-reads (10× Genomics, USA) library was prepared following the manufacturer’s instructions. The library was sequenced on the Illumina Nova-seq platform to generate 2 × 151-bp paired-end reads (Garvan Institute, Australia).

### Long-read library preparation and sequencing

Five long-read sequencing libraries for Oxford Nanopore MinION were prepared using the Ligation Sequencing Kit (SQK-LSK109) (Oxford Nanopore Technologies, Oxford, UK) following the manufacturer’s instructions. To increase the raw Nanopore read N50, the first and the second libraries were prepared using 1.75 and 0.75 µg of DNA extracted via a Circulomics kit from 2 adult male earwigs. Both libraries were sequenced in a single Minion flow cell, flushing the flow cell to remove remains of the first library before loading the second library with a Flow Cell Wash Kit (EXP-WSH004) (Oxford Nanopore Technologies, Oxford, UK).

To increase the total raw output, the third and the fourth libraries were prepared with DNA from 2 adult female earwigs, both extracted with a DNeasy Blood & Tissue, Qiagen kit followed by the AMPure XP beads clean-up step. Input DNA for these 2 libraries were 2.6 and 3.2 µg. These were each sequenced on an individual minion flow cell. The fifth library was prepared using 3.0 µg of DNA from an adult male earwig. As before DNA was extracted using a DNeasy Blood & Tissue, Qiagen kit followed by AMPure XP beads clean-up. However, before library preparation, the DNA was sheared 5 times using a 26 G × 0.5″ needle (Terumo, Japan). Since the sample type and the extraction method can impact the molecular weight of extracted DNA and the nanopore sequencing output, we tried samples from both sexes and different extraction protocols to optimize our sequencing output. All prepared libraries were sequenced with R9 chemistry MinION flow cell (FLO-MIN106) (Oxford Nanopore Technologies, UK) on a MinION connected to a laptop and operated with MinKNOW (v.2.0) interface.

### RNA extraction and sequencing

Total RNA from the different developmental stages, sex, and tissues was extracted using a Direct-zol RNA MicroPrep kit (Zymo Research, USA) with an on-filter DNAse treatment following the manufacturer’s instructions. Samples included: whole body (gut removed) of juvenile instars 1–2 and juvenile instars 3–4, dissected tissues (antennae, head, thorax, abdomen, legs, and gonads) of adult males and females. RNA from each individual and tissue type was extracted separately. RNA was quantified on a Qubit 2.0 Fluorometer (Life Technologies, USA) and initially quality checked using a nanodrop. Only high-quality extracts were further processed and were stored at −80°C until use.

RNA integrity was evaluated on a Fragment Analyzer (Advanced Analytical Technologies Inc., USA) at the Otago Genomics Facility (OGF), University of Otago, Dunedin, New Zealand. As with most of the insect RNA extracts (Winnebeck *et al.* 2010) RNA quality number (RQN) values ranged from 2.5 to 10 due to the collapsing of the 28S peak; quality was thus determined via the trace rather than RQN. Four pools of samples at equimolar concentration underwent library preparation. Pools consisted of: 8 whole body extractions for juvenile instar 1–2, 8 whole body extractions for juvenile instar 3–4, individual body tissues from 5 adult males, and individual body tissues from 5 adult females. TruSeq stranded mRNA libraries were prepared and sequenced as 2 × 100-bp paired-end reads across 2 lanes of HiSeq 2500 Rapid V2 flowcell at the OGF.

### Genome size estimation

Flow cytometry and *k*-mer-based approach with short-read data were used to estimate the genome size. Flow cytometry analysis was performed on a single head of earwig with 2 biological replicates at Flowjoanna (Palmerston North, NZ, USA). Briefly, the earwig’s head was dissociated with a pestle in 500 µl of the stock solution containing 0.1% w/v trisodium citrate dihydrate, 0.1% v/v IGEPAL, 0.052% w/v spermine tetrahydrochloride, and 0.006% sigma 7–9 (all Sigma-Aldrich, USA). Rooster red blood cells (RRBC) derived from the domestic chicken (*Gallus gallus*), stored in citrate buffer, were used as reference samples. Test samples were filtered through a 35-µl filter cap and further dissociated by adding 100 µl of 0.21 mg/ml trypsin followed by 75 µl of 2.5 mg/ml trypsin inhibitor (both Sigma-Aldrich) for 10 min at 37°C. Nuclei were stained using 100 µl of prestain (containing 416 mg/ml propidium iodide with 500 mg/ml RNAse in-stock solution). Two sample tubes, 1 prepared with RRBC and 1 prepared without, were then processed on a FACSCalibur (BD Biosciences, USA). The instrument was equipped with a 488-nm laser to produce fluorescence collected using the FL-2-Area signal (585/42 BP), along with forward scatter and side scatter signals that enabled RRBC nuclei to be resolved from earwig nuclei. Data were analyzed using Flowjo (BD Biosciences, USA) and the pg/nuclei of the sample calculated.

For *k*-mer-based genome size estimation, we used the paired-end linked-read sequences. Reads were processed with the scaff_reads script from Scaff10x (v.5.0) (https://github.com/wtsi-hpag/Scaff10X) to remove the 10× link adapters. Quality control was carried out with Trimmomatic (v.0.39) (Bolger *et al.* 2014) (options: SLIDINGWINDOW:4:15 LEADING:5 TRAILING:5 MINLEN:35). We used KMC (v.3.1.1) (Kokot *et al.* 2017) with a *k*-mer size of 21 to count the *k*-mers, the histogram produced was then visualized in Genomescope (v.2.0) (Ranallo-Benavidez *et al.* 2020) web browser.

### Phylogenetic analysis

Two sibling species of *F. auricularia* have been described (Wirth *et al.* 1998). To assess which of these we sequenced, nucleotide sequences covering the COI and COII region from 34 isolates of *F. auricularia* were downloaded from NCBI. Those included 15 isolates reported by Wirth *et al.* (1998) originally used to infer sibling species A and B and other isolates from Belgian orchards submitted to NCBI. Nucleotide sequence covering COI and COII regions from the assembled genome was extracted through BLAST hits. To ensure that a single subspecies was sequenced across all the individuals, raw reads from each run were blasted back to this sequence to ensure the presence of a single haplotype. The same genomic region extracted from the mitochondrial genome of *Euborellia arcanum* was used as an outgroup. Nucleotide sequences were aligned using Clustal Omega (v1.2.3) (Goujon *et al.* 2010). The evolutionary history was inferred using the Neighbor-Joining method (Saitou and Nei 1987) with 1,000 bootstrap replicates (Felsenstein 1985). The evolutionary distances were computed using the Maximum Composite Likelihood method (Tamura *et al.* 2004) and are in the units of the number of base substitutions per site. All ambiguous positions were removed for each nucleotide sequence pair (pairwise deletion option). There were a total of 799 positions in the final dataset. The optimal tree is presented and the evolutionary analyses were conducted in MEGA11 (Tamura *et al.* 2021).

### Bioinformatic pipeline

**Fig. 1.**
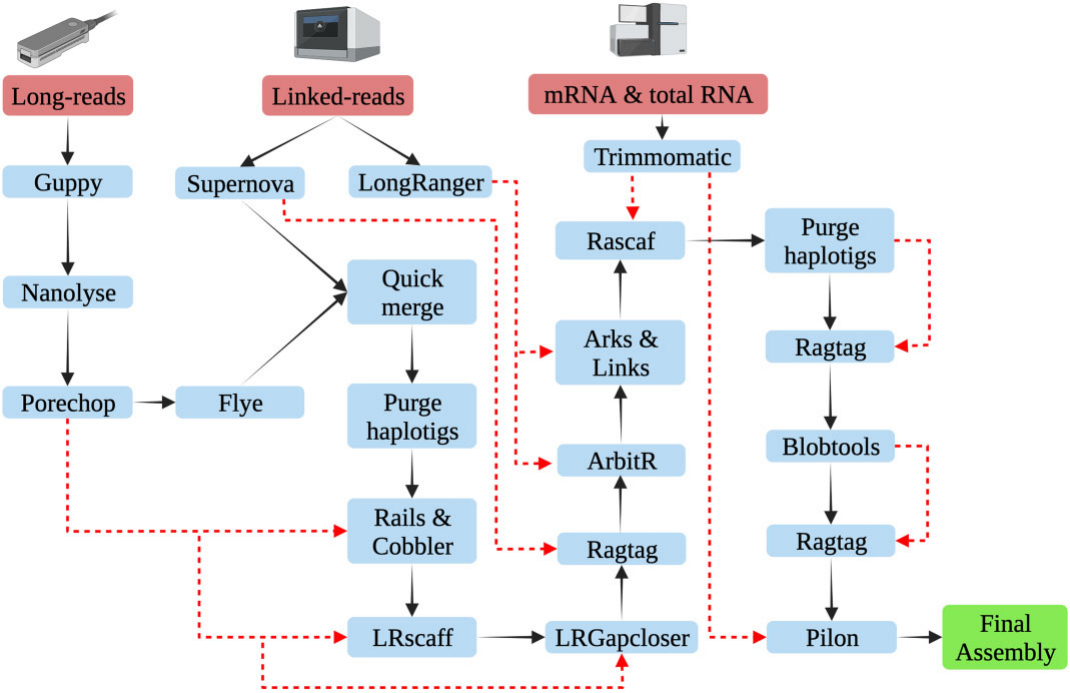
Schematic representation of the assembly pipeline for the *F. auricularia* genome. The solid black arrow represents the workflow and the red dotted lines represent the additional input data in the pipeline (created with BioRender.com).

All the scripts used for genome assembly, de novo repeat library construction, and annotation are available on GitHub (https://github.com/upendrabhattarai/Earwig_Genome_Project). The bioinformatics software and packages were run in New Zealand eScience Infrastructure. Below is a description of the pipeline (Fig. 1).

### Genome assembly

Paired-end Illumina reads from the Chromium library were assembled using Supernova (v.2.1.1) (Weisenfeld *et al.* 2017). Assembly metrics such as N50 values and contig/scaffold number were assessed using Quast (v.5.0.2) (Gurevich *et al.* 2013) and the presence of the single-copy ortholog genes was assessed using the insecta_odb10 database in BUSCO (v.5.1.3) (Simão *et al.* 2015). BUSCO score from Quast analysis wherever mentioned used BUSCO version 3.0.2 and the eukaryote_odb9 database. Based on several trial assemblies, we down-sampled the total input to 660 million paired-end reads using “—maxreads” option with “supernova run” to produce an assembly with better completeness and contiguity. The assembled fasta sequence was obtained with “pseudohap” style of the supernova “mkoutput” function.

Nanopore reads were basecalled using Guppy (v.5.0.7) (Wick *et al.* 2019) and processed with Nanolyse (v.1.2.0) (De Coster *et al.* 2018) and Porechop (v.0.2.4) (Wick *et al.* 2017) to remove lamda DNA and adapters from the raw reads. The reads were then assembled using Flye (v.2.7.1) (Kolmogorov *et al.* 2019) with default parameters. The Flye assembly had higher N50 and BUSCO scores compared to the Supernova assembly so we used the Flye assembly as a reference and supernova assembly as a query assembly in Quickmerge (v.0.3) (Chakraborty *et al.* 2016) to improve the contiguity and completeness of the assembly. The resulting assembly was processed with Purgehaplotigs (v.1.0.0) (Roach *et al.* 2018) to remove unpaired allelic contigs.

The purged genome underwent further scaffolding and gap-closing steps using Rails (v.1.5.1) and Cobbler (v.0.6.1) (Warren 2016), Lrscaf (v.1.1.11) (Qin *et al.* 2019), and Lrgapcloser (Xu *et al.* 2019) with the raw Nanopore long-read data. The resulting assembly was scaffolded with Ragtag (v.2.1.0) (Alonge *et al.* 2021) using the Supernova assembly. The raw linked-read data was aligned to the assembly with Long Ranger (v.2.0) (Ott *et al.* 2018) and used to further scaffold with ArbitR (v.0.2) (Hiltunen *et al.* 2021), Arks (v.1.0.4) (Coombe *et al.* 2018), and Links (v.1.8.7) (Warren *et al.* 2015). mRNA-seq reads sequenced for genome annotation purposes, and total RNA-seq reads sequenced for another project (manuscript under preparation) were also used for scaffolding the assembly with Rascaf (v.1.0.2) (Song *et al.* 2016). Duplicated and redundant haplotigs were again removed using Purgehaplotigs (Roach *et al.* 2018), and discarded haplotigs were used for scaffolding the assembly using Ragtag.

BlobTools2 (Laetsch and Blaxter 2017) was used to remove small (<1,000 bp) and low coverage contigs (<5× coverage). We followed the tutorial provided by the developers of the BlobTools2 in the genomehubs website for creating, updating, filtering, and generating plots (see more at: https://blobtoolkit.genomehubs.org/blobtools2/blobtools2-tutorials/). Contigs that were filtered out were used for re-scaffolding the assembly with Ragtag (v.1.0.2) (Alonge *et al.* 2021). Finally, we used 1 iteration of Pilon (v.1.24) (Walker *et al.* 2014) to polish the exonic region of the assembly using mRNA-seq data.

### Repeat content analysis

To assist with annotation a custom repeat library was generated for the Earwig genome using different de novo repeat and homology-based identifiers, including LTRharvest (v.1.5.10) (Ellinghaus *et al.* 2008), LTRdigest (v.1.5.10) (Steinbiss *et al.* 2009), RepeatModeler (v.2.0) (Flynn *et al.* 2020), TransposonPSI (v.1.0.0) (Haas 2010), and SINEBase (v.1.1) (Vassetzky and Kramerov 2013). We concatenated the individual libraries, and sequences with more than 80% similarity were merged to remove redundancy using usearch (v.11.0.667) (Edgar 2010). The library was then classified with RepeatClassifier (v.2.0) (Flynn *et al.* 2020). Sequences with unknown categories in the library were mapped against the UniProtKB/Swiss-Prot database (*e*-value <1e−01); if sequences were not annotated as repeat sequences they were removed from the library. The final repeat library was used in RepeatMasker (v.4.1.2) (Chen 2004) to generate a report for genome repeat content and provided to the MAKER2 pipeline to mask the genome.

### Genome annotation

Genome annotation was carried out with 3 iterations of the MAKER2 (v.2.31.9) (Holt and Yandell 2011) pipeline combining evidence-based and ab initio gene models. The first round of MAKER2 used evidence-based models and the other 2 rounds were run using ab initio gene models. For the first round, we provided the MAKER2 pipeline with 180,119 mRNA transcripts *denovo* assembled via the Trinity pipeline (v.2.13.2) (Grabherr *et al.* 2011) along with 26,414 mRNA and 1,529 protein sequences of dermapterans from NCBI and 779 dermapteran protein sequences from the Uniprot database.

Augustus was trained using BRAKER (v.2.16) (Hoff *et al.* 2019) and SNAP was trained after each round of MAKER2 to use for ab initio gene model prediction. For the functional annotation, we ran InterProScan (v.5.51-85.0) (Jones *et al.* 2014) for the predicted protein sequences obtained from MAKER2 and retrieved InterPro ID, PFAM domains, and Gene Ontology (GO) terms. Furthermore, we ran BLASTp (Altschul *et al.* 1990) with the Uniprot database to assign gene descriptors to each transcript based on the best BLAST hit.

## Results and discussion

### Genome size estimates

The flow cytometer estimated the genome size of 968.22 ± 20.747 Mb (mean ± SD) for the earwig genome. Similarly, the *k*-mer-based approach using adapter removed paired-end data from linked-read sequencing estimated the male earwig to be 988 Mb. Whereas an earlier estimation of an unknown dermapteran (earwig) species genome size was 1.4 Gb (Gregory 2005) showing a variable genome size within the order.

### Phylogenetic analysis

**Fig. 2.**
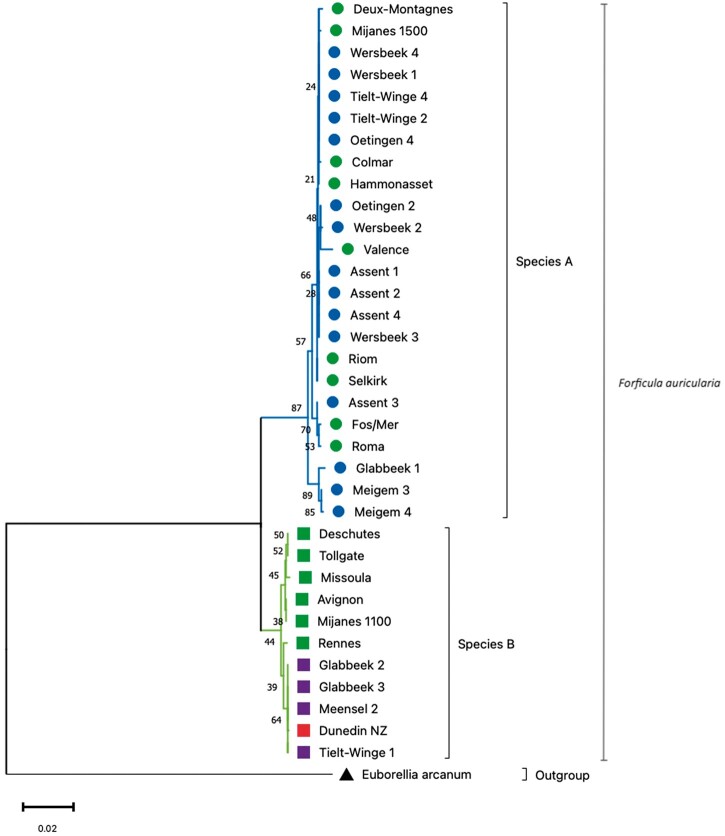
The phylogenetic relationships of *F. auricularia* obtained from different geographic regions inferred from COI and COII using a Neighbour-Joining method and Maximum Composite Likelihood approach in MEGA11*.* All ambiguous positions were removed for each nucleotide sequence pair (pairwise deletion). The percentage of replicate trees in which the associated taxa clustered together in the bootstrap test (1,000 replicates) are shown next to the branches. The tree is drawn to scale, with branch lengths in the same units as those of the evolutionary distances used to infer the phylogenetic tree. Species labeled with the colored squares are subspecies B. The red square (Dunedin NZ) is the one for which the genome is reported in this article. Green squares are the species categorized as subspecies B by Wirth *et al.* (1998) and the purple squares are others for which the nucleotide sequences were downloaded from NCBI. Species labeled with colored circles belong to subspecies A. Green circles represent subspecies A inferred by Wirth *et al.* (1998) and blue are other species for which nucleotide sequences were downloaded from NCBI. *E. arcanum* is the outgroup labelled with a black triangle.

The phylogenetic analysis showed 2 distinct subspecies groups within *F. auricularia* (Fig. 2), in agreement with Wirth *et al.* (1998). One clade includes 24 individuals including 9 originally identified as species A (green circle labels, Fig. 2). While the remaining 11 individuals cluster into a separate clade that include the assembled genome herein (red square label, Fig. 2) and 6 individuals originally identified as species B (green square labels, Fig. 2). The analysis confirmed that the genome reported in this article (Dunedin, NZ) belongs to the subspecies B of *F. auricularia*. This is also in accordance with the report from Quarrell *et al.* (2018) where 2 isolates from New Zealand were reported as subspecies B of *F. auricularia*.

### Genome assembly

A total of 799.6 million paired-end reads was generated from 10× linked-read sequencing. Downsampled to 660 million paired-end reads, Supernova estimated the genome size of 1.22 Gb, raw coverage of 82.02%, effective coverage of 39.50% and weighted mean molecule size of 22.45 kb. The Supernova assembly was 1.15 Gb in size and had 145,055 contigs, with an N50 of 0.03 Mb and L50 of 7,500. Quast reported a complete BUSCO of 64.69% and a partial BUSCO of 9.24% from the eukaryotic database.

The Nanopore sequencing yielded approximately 10.7 Gb of data, consisting of over 3 million reads. The median read length was 897 bp with an N50 length of 11,986 bp (Supplementary Table 1). The median read Phred quality was 13.34. Flye produced an assembly of 1.1 Gb, comprised of 18,766 contigs with N50 of 0.18 Mb and L50 of 1,832. Quast reported a complete BUSCO of 82.18% and a partial BUSCO of 9.24%. The long-read assembly was more complete based on the BUSCO scores and demonstrated better contiguity, so we merged the 2 assemblies using the Flye assembly as the primary assembly (Table 1).

**Table 1.** Assembly statistics at different stages of assembly for the genome of the European earwig *F. auricularia.*

	Assembly length	No. scaffolds	N50	L50	Ns per 100 kbp	BUSCO % (Quast)	
						Complete	Partial
Supernova assembly	1,145,470,221	145,055	30,358	7,500	3,677.89	64.69	9.24
Flye assembly	1,118,374,848	18,766	180,737	1,832	0.35	82.18	9.24
Final hybrid assembly	1,062,210,345	919	12,548,649	20	846.85	87.13	2.97

The Supernova and the Flye assembly statistics are for the assembly right after the assembler and no further processing, whereas the Final hybrid assembly shows the statistics of the assembly through all the assembly process as described in this article. Quast scores are to its default Eukaryota database.

The BlobTools2 filtering produced a clean assembly with only 215 contigs out of 2.7 K assigned as no-hits and all other contigs with blast hits to the Arthropoda database (Supplementary Fig. 1).The final hybrid assembly has a size of 1.06 Gb. It has 919 scaffolds with an N50 of 12.55 Mb, which shows that the assembly is highly contiguous. Half of the genome is present in just 20 scaffolds, as denoted by the L50 number (Table 1). Assembly has 846.85 “N’s” per 100kbp. The BUSCO score from the insect database (*n* = 1,367) for the assembly is 87.1% complete, among which 4.1% were duplicated, and 3.1% fragmented BUSCO (Supplementary Fig. 2). Improvement in assembly statistics after each processing step is given in Supplementary Table 2.

The only other whole-genome sequence publicly available from the Dermaptera order is of the earwig *Anisolabis maritima* [GenBank assembly accession: GCA_010014785.1, available to download from InsectBase (v.2)]. The *A. maritima* genome assembly is 649.7 Mb with a N50 of 1.4 Mb, (Mei *et al.* 2022), while its BUSCO score is 83.4% complete and 10.8% fragmented using the insect database (*n* = 1,367). In comparison, the *F. auricularia* genome assembly has a better gene model and contiguity.

### Genome repeat contents

Repeat analysis of the assembly showed that interspersed repeats comprised 686.43 Mb (64.62%) of the *F. auricularia* genome. This includes 248.24 Mb of retroelements (23.37% of the genome), 178.33 Mb of DNA transposons (16.79% of the genome), 35.83 Mb of rolling circles (3.28% of the genome) and 260.87 Mb of unclassified elements (Table 2). Unusually large and variable genome sizes characterize Hemimetabolans (Wu *et al.* 2017). Comparative analysis in 6 species of Gomphocerine grasshoppers showed a strong positive correlation between repeat content and genome size. Genome size ranged from 8.2 to 13.7 Gb in these 6 species with a repeat content ranging from 79% to 87%, with the exception of *Stauroderus scalaris* whose genome is 96% repetitive DNA and the second-largest insect genome documented. Our estimation of genome size for *F. auricularia* does not show gigantism (968.22 Mb, flow cytometer estimate). However, its repeatome (64.62%) is almost twice that of other hemimetabolous insects like *Gryllus bimaculatus* (33.69%) and *Laupala kohalensis* (35.51%) (Ylla *et al.* 2021). This fold increase in the repeatome is surprising given both *G. bimaculatus* and *L. kohalensis* have bigger genomes (1.6 Gb) than *F. auricularia*.

**Table 2.** Repeat content analysis in the European earwig *Forficula auricularia* genome.

No. sequences	919		
Total length (bp)	1,062,210,345		
GC level	31.03%		
Bases masked	722,769,501 bp (68.04%)		
	Numbers	Length (bp)	Percentage
Retroelements	1,385,007	248,236,495	23.37
SINEs	41,157	5,138,497	0.48
Penelope	50,409	10,372,837	0.98
LINEs	660,178	124,985,146	11.77
CRE/SLACS	0	0	0.00
L2/CR1/Rex	112,418	20,654,321	1.94
R1/LOA/Jockey	167,317	22,277,052	2.10
R2/R4/NeSL	23,348	4,271,189	0.40
RTE/Bov-B	136,406	28,799,096	2.71
L1/CIN4	10,079	1,892,539	0.18
LTR elements	683,672	118,112,852	11.12
BEL/Pao	60,561	12,114,300	1.14
Ty1/Copia	97,132	14,352,992	1.35
Gypsy/DIRS1	521,467	91,083,363	8.57
Retroviral	3,701	443,583	0.04
DNA transposons	1,040,870	178,326,460	16.79
hobo-Activator	362,395	59,188,939	5.57
Tc1-IS630-Pogo	355,781	66,331,225	6.24
En-Spm	0	0	0.00
MuDR-IS905	0	0	0.00
PiggyBac	21,153	2,726,812	0.26
Tourist/Harbinger	5,541	1,187,174	0.11
Other (Mirage, P-element, Transib)	10,240	1,580,945	0.15
Rolling circles	174,964	34,830,487	3.28
Unclassified	1,563,937	259,874,747	24.47
Total interspersed repeats		686,437,702	64.62
Small RNA	9,913	1,406,877	0.13
Satellites	1,110	495,561	0.05
Simple repeats	0	0	0.00
Low complexity	0	0	0.00

### Genome annotation

Combining evidence-based and ab initio gene models in the MAKER2 pipeline, we identified 12,876 genes and 21,031 mRNAs in the genome assembly. The mean gene length is 12,096 bp and the total gene length is 155.75 Mb, which makes 14.7% of the whole assembly. The longest gene annotated is 412,198 bp and the longest CDS is 19,035 bp (Table 3). 61.35% of total predicted mRNAs and 59.53% of predicted proteins are also functionally annotated through either 1 or more of InterPro, GO, and Pfam databases (Supplementary Table 3). The annotated transcriptome and proteome had a complete BUSCO score of 73.4% and 70% respectively using the insect database (Supplementary Fig. 3). 98.3% of the gene models have AED score of 0.5 or less, assuring highly confident gene prediction (Supplementary Fig. 4).

**Table 3.** Genome annotation summary for the European earwig *Forficula auricularia*.

Total sequence length	1,062,210,345
Number of genes	12,876
Number of mRNAs	21,031
Number of exons	145,003
Number of introns	123,973
Number of CDS	21,030
Total gene length	155,753,058
Total mRNA length	271,884,000
Total exon length	32,584,454
Total intron length	239,538,939
Total CDS length	23,936,568
Longest gene	412,198
Longest mRNA	412,198
Longest exon	10,240
Longest intron	319,382
Longest CDS	19,035
Mean gene length	12,096
Mean mRNA length	12,928
Mean exon length	225
Mean intron length	1,932
Mean CDS length	1,138
% of genome covered by genes	14.7
% of genome covered by CDS	2.3
Mean mRNAs per gene	2
Mean exons per mRNA	7
Mean introns per mRNA	6

**Fig. 3.**
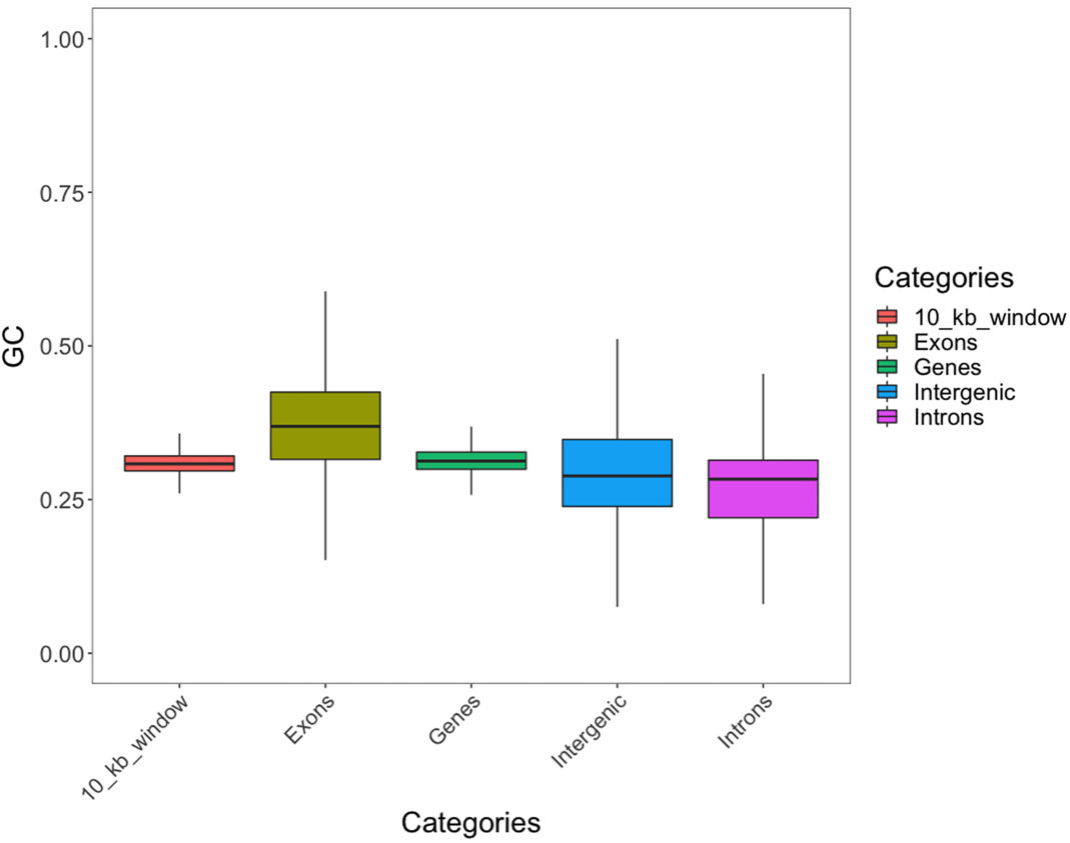
GC percentage in different genomic features of the *F. auricularia* genome. GC content for 10-kb windows was generated without regard to any genomic features. Whiskers extend to 25th and 75th percentiles. GC content in exons is higher and in introns is lower compared to the genome average.

The GC content of the *F. auricularia* genome is 31.03%, far greater than the 19.3% GC in the genome of the earwig *A. maritima* reported in InsectBase2 database (Mei *et al.* 2022). So we compared the GC content between different regions of *F. auricularia* genome to see if there are any abnormal distributions. Our analysis showed that exons have higher GC content (0.372 ± 0.087) (mean ± SD) and introns have lower (0.267 ± 0.075) when compared between intergenic regions (*N* = 823,037), genes (*N* = 12,876), exons (*N* = 145,003), introns (*N* = 123,973), and nonoverlapping 10-kb windows throughout the genome (*N* = 106,686) (Fig. 3). GC content for 10-kb windows was 0.308 ± 0.032, which resembles the mean GC content of the whole genome (0.310). This finding is not unexpected as a higher GC content in exons vs. introns is common across the animal and plant kingdom because of the evolutionary selection of exon regions (Amit *et al.* 2012). There was a significant difference for each pairwise comparison using ANOVA followed by Tukey HSD with *P < *0.0001.

Recently there has been a growing interest in hemimetabolous insects for use as genetic research models and hence sequencing and analyzing their genomes (Adamski *et al.* 2019; Ylla *et al.* 2021). Because of their primitive yet successful biology, the evolutionary insights they can offer for various biological traits are enormous. Genomes of milkweed bug (*Oncopeltus fasciatus*) (Panfilio *et al.* 2019) and field cricket (*G. bimaculatus*) (Ylla *et al.* 2021) have been instrumental for developmental biology research. Similarly, the genome of *Rhodnius prolixus*, a medically important hemimetabolous insect vector, provides key insights into the genetic re-organization contributing to the evolution of a blood-feeding lifestyle (Mesquita *et al.* 2015). Furthermore, the genome of *Halyomorpha halys* has informed research on polyphagy and insecticide resistance and contributed to advances in research on insect–pest control strategies (Sparks *et al.* 2020). In this context, we believe that, the genome of *F. auricularia* will be a key resource to develop this important insect species as a genetic model. We anticipate this will enhance the genetic study on various aspects of its biology, including developmental biology, sociality, and evolutionary characteristics.

## Supplementary Material

jkac199_Supplementary_Data

## Data Availability

The genome assembly and annotation of *F. auricularia* are available through FigShare https://doi.org/10.6084/m9.figshare.19092044. The raw sequencing reads are deposited in NCBI with accession number PRJNA800435. The scripts used for genome assembly, repeat library preparation and masking, and genome annotation are available at GitHub under GNU GPLv3 license (https://github.com/upendrabhattarai/Earwig_genome_project). Supplemental material is available at *G3* online.

## References

[jkac199-B1] Adamski Z , Bufo SA, Chowański S, Falabella P, Lubawy J, Marciniak P, Pacholska-Bogalska J, Salvia R, Scrano L, Słocińska M, et al Beetles as model organisms in physiological, biomedical and environmental studies—a review. Front Physiol. 2019;10:319. 30984018 10.3389/fphys.2019.00319 PMC6447812

[jkac199-B2] Alonge M , Lebeigle L, Kirsche M, Aganezov S, Wang X, Lippman ZB, Schatz MC, Soyk S. Automated assembly scaffolding elevates a new tomato system for high-throughput genome editing. bioRxiv 2021.11.18.469135, 2021. 10.1101/2021.11.18.469135 PMC9753292 36522651

[jkac199-B3] Altschul SF , Gish W, Miller W, Myers EW, Lipman DJ. Basic local alignment search tool. J Mol Biol. 1990;215(3):403–410. 2231712 10.1016/S0022-2836(05)80360-2

[jkac199-B4] Amit M , Donyo M, Hollander D, Goren A, Kim E, Gelfman S, Lev-Maor G, Burstein D, Schwartz S, Postolsky B, et al Differential GC content between exons and introns establishes distinct strategies of splice-site recognition. Cell Rep. 2012;1(5):543–556. 22832277 10.1016/j.celrep.2012.03.013

[jkac199-B5] Binns M , Hoffmann AA, van Helden M, Heddle T, Hill MP, Macfadyen S, Nash MA, Umina PA. Lifecycle of the invasive omnivore, *Forficula auricularia*, in Australian grain growing environments. Pest Manag Sci. 2021;77(4):1818–1828. 33274578 10.1002/ps.6206 PMC7986395

[jkac199-B6] Bolger AM , Lohse M, Usadel B. Trimmomatic: a flexible trimmer for Illumina sequence data. Bioinformatics. 2014;30(15):2114–2120. 24695404 10.1093/bioinformatics/btu170 PMC4103590

[jkac199-B7] Campos MR , Picanço MC, Martins JC, Tomaz AC, Guedes RNC. Insecticide selectivity and behavioral response of the earwig *Doru luteipes*. Crop Prot. 2011;30(12):1535–1540.

[jkac199-B8] Chakraborty M , Baldwin-Brown JG, Long AD, Emerson JJ. Contiguous and accurate de novo assembly of metazoan genomes with modest long read coverage. Nucleic Acids Res. 2016;44:gkw654. 10.1093/nar/gkw654 PMC5100563 27458204

[jkac199-B9] Chen N. Using RepeatMasker to identify repetitive elements in genomic sequences. Curr Protoc Bioinforma. 2004;Chapter 4:Unit 4.10. 10.1002/0471250953.bi0410s05 18428725

[jkac199-B10] Coombe L , Zhang J, Vandervalk BP, Chu J, Jackman SD, Birol I, Warren RL. ARKS: chromosome-scale scaffolding of human genome drafts with linked read kmers. BMC Bioinformatics. 2018;19(1):234. 29925315 10.1186/s12859-018-2243-x PMC6011487

[jkac199-B11] De Coster W , D'Hert S, Schultz DT, Cruts M, Van Broeckhoven C. NanoPack: visualizing and processing long-read sequencing data. Bioinformatics. 2018;34(15):2666–2669. 29547981 10.1093/bioinformatics/bty149 PMC6061794

[jkac199-B12] Edgar RC. Search and clustering orders of magnitude faster than BLAST. Bioinformatics. 2010;26(19):2460–2461. 20709691 10.1093/bioinformatics/btq461

[jkac199-B13] Ellinghaus D , Kurtz S, Willhoeft U. LTRharvest, an efficient and flexible software for de novo detection of LTR retrotransposons. BMC Bioinformatics. 2008;9:18. 18194517 10.1186/1471-2105-9-18 PMC2253517

[jkac199-B14] Falk J , Wong JWY, Kölliker M, Meunier J. Sibling cooperation in earwig families provides insights into the early evolution of social life. Am Nat. 2014;183(4):547–557. 24642498 10.1086/675364

[jkac199-B15] Felsenstein J. Confidence limits on phylogenies: an approach using the bootstrap. Evolution (N Y). 1985;39(4):783. 10.1111/j.1558-5646.1985.tb00420.x 28561359

[jkac199-B16] Flynn JM , Hubley R, Goubert C, Rosen J, Clark AG, Feschotte C, Smit AF. RepeatModeler2 for automated genomic discovery of transposable element families. Proc Natl Acad Sci U S A. 2020;117(17):9451–9457. 32300014 10.1073/pnas.1921046117 PMC7196820

[jkac199-B17] Forslund P. Male–male competition and large size mating advantage in European earwigs, *Forficula auricularia*. Anim Behav. 2000;59(4):753–762. 10792930 10.1006/anbe.1999.1359

[jkac199-B18] Goujon M , McWilliam H, Li W, Valentin F, Squizzato S, Paern J, Lopez R. A new bioinformatics analysis tools framework at EMBL–EBI. Nucleic Acids Res. 2010;38(Web Server issue):W695–W699. 20439314 10.1093/nar/gkq313 PMC2896090

[jkac199-B19] Grabherr MG , Haas BJ, Yassour M, Levin JZ, Thompson DA, Amit I, Adiconis X, Fan L, Raychowdhury R, Zeng Q, et al Full-length transcriptome assembly from RNA-Seq data without a reference genome. Nat Biotechnol. 2011;29(7):644–652. 21572440 10.1038/nbt.1883 PMC3571712

[jkac199-B20] Gregory TR. Genome size evolution in animals. In: Gregory TR, editor. The Evolution of the Genome. Burlington: Elsevier; 2005. p. 3–87.

[jkac199-B21] Guillet S , Guiller A, Deunff J, Vancassel M. Analysis of a contact zone in the *Forficula auricularia* L. (Dermaptera: Forficulidae) species complex in the Pyrenean Mountains. Heredity (Edinb). 2000; 85(5):444–449. 11122422 10.1046/j.1365-2540.2000.00775.x

[jkac199-B22] Guillet S , Josselin N, Vancassel M. Multiple introductions of the *Forficula auricularia* species complex (Dermaptera: Forficulidae) in eastern North America. Can Entomol. 2000; 132(1):49–57.

[jkac199-B23] Gurevich A , Saveliev V, Vyahhi N, Tesler G. QUAST: quality assessment tool for genome assemblies. Bioinformatics. 2013;29(8):1072–1075. 23422339 10.1093/bioinformatics/btt086 PMC3624806

[jkac199-B24] Haas B. TransposonPSI: An Application of PSI-Blast to Mine (Retro-)Transposon ORF Homologies; Cambridge, MA, USA: Broad Institute, 2010.

[jkac199-B25] Herbison REH , Evans S, Doherty J-F, Poulin R. Let’s go swimming: mermithid-infected earwigs exhibit positive hydrotaxis. Parasitology. 2019;146(13):1631–1635. 31397259 10.1017/S0031182019001045

[jkac199-B26] Hill MP , Binns M, Umina PA, Hoffmann AA, Macfadyen S. Climate, human influence and the distribution limits of the invasive European earwig, *Forficula auricularia*, in Australia. Pest Manag Sci. 2019;75(1):134–143. 30168641 10.1002/ps.5192

[jkac199-B27] Hiltunen M , Ryberg M, Johannesson H. ARBitR: an overlap-aware genome assembly scaffolder for linked reads. Bioinformatics. 2021;37(15):2203–2205. 33216122 10.1093/bioinformatics/btaa975 PMC8352505

[jkac199-B28] Hoff KJ , Lomsadze A, Borodovsky M, Stanke M. Whole-genome annotation with BRAKER. Methods Mol Biol. 2019;1962:65–95. 31020555 10.1007/978-1-4939-9173-0_5 PMC6635606

[jkac199-B29] Holt C , Yandell M. MAKER2: an annotation pipeline and genome-database management tool for second-generation genome projects. BMC Bioinformatics. 2011;12:491. 22192575 10.1186/1471-2105-12-491 PMC3280279

[jkac199-B30] Jones P , Binns D, Chang H-Y, Fraser M, Li W, McAnulla C, McWilliam H, Maslen J, Mitchell A, Nuka G, et al InterProScan 5: genome-scale protein function classification. Bioinformatics. 2014;30(9):1236–1240. 24451626 10.1093/bioinformatics/btu031 PMC3998142

[jkac199-B31] Kamimura Y. Right-handed penises of the earwig *Labidura riparia* (Insecta, Dermaptera, Labiduridae): evolutionary relationships between structural and behavioral asymmetries. J Morphol. 2006;267(11):1381–1389. 17051546 10.1002/jmor.10484

[jkac199-B32] Kamimura Y , Matsumura Y, Yang C-CS, Gorb SN. Random or handedness? Use of laterally paired penises in Nala earwigs (Insecta: Dermaptera: Labiduridae). Biol J Linn Soc. 2021;134(3):716–731.

[jkac199-B33] Kokot M , Dlugosz M, Deorowicz S. KMC 3: counting and manipulating k-mer statistics. Bioinformatics. 2017;33(17):2759–2761. 28472236 10.1093/bioinformatics/btx304

[jkac199-B34] Kolmogorov M , Yuan J, Lin Y, Pevzner PA. Assembly of long, error-prone reads using repeat graphs. Nat Biotechnol. 2019;37(5):540–546. 30936562 10.1038/s41587-019-0072-8

[jkac199-B35] Kramer J , Thesing J, Meunier J. Negative association between parental care and sibling cooperation in earwigs: a new perspective on the early evolution of family life? J Evol Biol. 2015;28(7):1299–1308. 25975926 10.1111/jeb.12655

[jkac199-B36] Labandeira CC , Phillips TL. A Carboniferous insect gall: insight into early ecologic history of the Holometabola. Proc Natl Acad Sci U S A. 1996;93(16):8470–8474. 11607697 10.1073/pnas.93.16.8470 PMC38695

[jkac199-B37] Laetsch DR , Blaxter ML. BlobTools: interrogation of genome assemblies. F1000Research. 2017;6:1287.

[jkac199-B38] Lamb RJ , Wellington WG. Techniques for studying the behavior and ecology of the European earwig, Forficula auricularia (Dermaptera: Forficulidae). Can Entomol. 1974;106(8):881–888.

[jkac199-B39] Mei Y , Jing D, Tang S, Chen X, Chen H, Duanmu H, Cong Y, Chen M, Ye X, Zhou H, et al InsectBase 2.0: a comprehensive gene resource for insects. Nucleic Acids Res. 2022;50(D1):D1040–D1045. 34792158 10.1093/nar/gkab1090 PMC8728184

[jkac199-B40] Mesquita RD , Vionette-Amaral RJ, Lowenberger C, Rivera-Pomar R, Monteiro FA, Minx P, Spieth J, Carvalho AB, Panzera F, Lawson D, et al Genome of *Rhodnius prolixus*, an insect vector of Chagas disease, reveals unique adaptations to hematophagy and parasite infection. Proc Natl Acad Sci U S A. 2015;112(48):14936–14941. 26627243 10.1073/pnas.1506226112 PMC4672799

[jkac199-B41] Van Meyel S , Meunier J. Filial egg cannibalism in the European earwig: its determinants and implications in the evolution of maternal egg care. Anim Behav. 2020;164:155–162.

[jkac199-B42] Mukherjee K , Twyman RM, Vilcinskas A. Insects as models to study the epigenetic basis of disease. Prog Biophys Mol Biol. 2015;118(1–2):69–78. 25778758 10.1016/j.pbiomolbio.2015.02.009

[jkac199-B43] Ott A , Schnable JC, Yeh C-T, Wu L, Liu C, Hu H-C, Dalgard CL, Sarkar S, Schnable PS. Linked read technology for assembling large complex and polyploid genomes. BMC Genomics. 2018;19(1):651. 30180802 10.1186/s12864-018-5040-z PMC6122573

[jkac199-B44] Panfilio KA , Vargas Jentzsch IM, Benoit JB, Erezyilmaz D, Suzuki Y, Colella S, Robertson HM, Poelchau MF, Waterhouse RM, Ioannidis P, et al Molecular evolutionary trends and feeding ecology diversification in the Hemiptera, anchored by the milkweed bug genome. Genome Biol. 2019;20(1):64. 30935422 10.1186/s13059-019-1660-0 PMC6444547

[jkac199-B45] Qin M , Wu S, Li A, Zhao F, Feng H, Ding L, Ruan J. LRScaf: improving draft genomes using long noisy reads. BMC Genomics. 2019;20(1):955. 31818249 10.1186/s12864-019-6337-2 PMC6902338

[jkac199-B46] Quarrell SR , Arabi J, Suwalski A, Veuille M, Wirth T, Allen GR. The invasion biology of the invasive earwig, *Forficula auricularia* in Australasian ecosystems. Biol Invasions. 2018;20(6):1553–1565.

[jkac199-B47] Ranallo-Benavidez TR , Jaron KS, Schatz MC. GenomeScope 2.0 and Smudgeplot for reference-free profiling of polyploid genomes. Nat Commun. 2020;11(1):1432. 32188846 10.1038/s41467-020-14998-3 PMC7080791

[jkac199-B48] Roach MJ , Schmidt SA, Borneman AR. Purge Haplotigs: allelic contig reassignment for third-gen diploid genome assemblies. BMC Bioinformatics. 2018;19(1):460. 30497373 10.1186/s12859-018-2485-7 PMC6267036

[jkac199-B49] Saitou N , Nei M. The neighbor-joining method: a new method for reconstructing phylogenetic trees. Mol Biol Evol. 1987;4(4):406–425. 3447015 10.1093/oxfordjournals.molbev.a040454

[jkac199-B50] Simão FA , Waterhouse RM, Ioannidis P, Kriventseva EV, Zdobnov EM. BUSCO: assessing genome assembly and annotation completeness with single-copy orthologs. Bioinformatics. 2015;31(19):3210–3212. 26059717 10.1093/bioinformatics/btv351

[jkac199-B51] Simons M , Tibbetts E. Insects as models for studying the evolution of animal cognition. Curr Opin Insect Sci. 2019;34:117–122. 31271948 10.1016/j.cois.2019.05.009

[jkac199-B52] Song L , Shankar DS, Florea L. Rascaf: improving genome assembly with RNA sequencing data. Plant Genome. 2016;9. doi: 10.3835/plantgenome2016.03.0027. 27902792

[jkac199-B53] Sparks ME , Bansal R, Benoit JB, Blackburn MB, Chao H, Chen M, Cheng S, Childers C, Dinh H, Doddapaneni HV, et al Brown marmorated stink bug, *Halyomorpha halys* (Stål), genome: putative underpinnings of polyphagy, insecticide resistance potential and biology of a top worldwide pest. BMC Genomics. 2020;21(1):227. 32171258 10.1186/s12864-020-6510-7 PMC7071726

[jkac199-B54] Steinbiss S , Willhoeft U, Gremme G, Kurtz S. Fine-grained annotation and classification of de novo predicted LTR retrotransposons. Nucleic Acids Res. 2009;37(21):7002–7013. 19786494 10.1093/nar/gkp759 PMC2790888

[jkac199-B55] Tamura K , Nei M, Kumar S. Prospects for inferring very large phylogenies by using the neighbor-joining method. Proc Natl Acad Sci U S A. 2004;101(30):11030–11035. 15258291 10.1073/pnas.0404206101 PMC491989

[jkac199-B56] Tamura K , Stecher G, Kumar S. MEGA11: Molecular Evolutionary Genetics Analysis Version 11. Mol Biol Evol. 2021;38(7):3022–3027. 33892491 10.1093/molbev/msab120 PMC8233496

[jkac199-B57] Tourneur J-C , Meunier J. Thermal regimes, but not mean temperatures, drive patterns of rapid climate adaptation at a continent-scale: evidence from the introduced European earwig across North America. bioRxiv 550319, 2019. 10.1101/550319

[jkac199-B58] Truman JW. The evolution of insect metamorphosis. Curr Biol. 2019;29(23):R1252–R1268. 31794762 10.1016/j.cub.2019.10.009

[jkac199-B59] Vassetzky NS , Kramerov DA. SINEBase: a database and tool for SINE analysis. Nucleic Acids Res. 2013;41(Database issue):D83–D89. 23203982 10.1093/nar/gks1263 PMC3531059

[jkac199-B60] Walker BJ , Abeel T, Shea T, Priest M, Abouelliel A, Sakthikumar S, Cuomo CA, Zeng Q, Wortman J, Young SK, et al Pilon: an integrated tool for comprehensive microbial variant detection and genome assembly improvement. PLoS One. 2014;9(11):e112963. 25409509 10.1371/journal.pone.0112963 PMC4237348

[jkac199-B61] Warren RL. RAILS and Cobbler: scaffolding and automated finishing of draft genomes using long DNA sequences. JOSS. 2016;1(7):116.

[jkac199-B62] Warren RL , Yang C, Vandervalk BP, Behsaz B, Lagman A, Jones SJM, Birol I. LINKS: scalable, alignment-free scaffolding of draft genomes with long reads. Gigascience. 2015;4:35. 26244089 10.1186/s13742-015-0076-3 PMC4524009

[jkac199-B63] Weisenfeld NI , Kumar V, Shah P, Church DM, Jaffe DB. Direct determination of diploid genome sequences. Genome Res. 2017;27(5):757–767. 28381613 10.1101/gr.214874.116 PMC5411770

[jkac199-B64] Wick RR , Judd LM, Gorrie CL, Holt KE. Completing bacterial genome assemblies with multiplex MinION sequencing. Microb. genomics. 2017;3(10):e000132. e000132. 10.1099/mgen.0.000132 PMC5695209 29177090

[jkac199-B65] Wick RR , Judd LM, Holt KE. Performance of neural network basecalling tools for Oxford Nanopore sequencing. Genome biol. 2019;20:129. https://doi.org/10.1186/s13059-019-1727-y. 10.1186/s13059-019-1727-y PMC6591954 31234903

[jkac199-B66] Wilson-Sanders SE. Invertebrate models for biomedical research, testing, and education. ILAR J. 2011;52(2):126–152. 21709307 10.1093/ilar.52.2.126

[jkac199-B67] Winnebeck EC , Millar CD, Warman GR. Why does insect RNA look degraded? J Insect Sci. 2010;10(159):1–7. 21067419 10.1673/031.010.14119 PMC3016993

[jkac199-B68] Wirth T , Le Guellec R, Vancassel M, Veuille M. Molecular and reproductive characterization of sibling species in the European earwig (*Forficula auricularia*). Evolution (N Y). 1998;52(1):260. 10.1111/j.1558-5646.1998.tb05160.x 28568161

[jkac199-B69] Wu C , Twort VG, Crowhurst RN, Newcomb RD, Buckley TR. Assembling large genomes: analysis of the stick insect (*Clitarchus hookeri)* genome reveals a high repeat content and sex-biased genes associated with reproduction. BMC Genomics. 2017;18(1):884. 29145825 10.1186/s12864-017-4245-x PMC5691397

[jkac199-B70] Xu G-C , Xu T-J, Zhu R, Zhang Y, Li S-Q, Wang H-W, Li J-T. LR_Gapcloser: a tiling path-based gap closer that uses long reads to complete genome assembly. Gigascience. 2019;8(1):10.1093/gigascience/giy157 PMC6324547 30576505

[jkac199-B71] Yang AS. Modularity, evolvability, and adaptive radiations: a comparison of the hemi- and holometabolous insects. Evol Dev. 2001;3(2):59–72. 11341675 10.1046/j.1525-142x.2001.003002059.x

[jkac199-B72] Ylla G , Nakamura T, Itoh T, Kajitani R, Toyoda A, Tomonari S, Bando T, Ishimaru Y, Watanabe T, Fuketa M, et al Insights into the genomic evolution of insects from cricket genomes. Commun Biol. 2021;4(1):733. 34127782 10.1038/s42003-021-02197-9 PMC8203789

