# Supplementary material for: Genome assembly and annotation of the European earwig Forficula auricularia (subspecies B)
Source: G3 (Bethesda). 2022 Aug 16;12(10):jkac199. doi: 10.1093/g3journal/jkac199 (PMC9526046; doi:10.1093/g3journal/jkac199)
Supplement: jkac199_Supplementary_Data [file jkac199_supplementary_data.pdf]

Supplementary file

Genome assembly and annotation of the European earwig *Forficula auricularia* (subspecies B).

Upendra R. Bhattarai[1], Mandira Katuwal[1], Robert Poulin[2], Neil J. Gemmell[1*], Eddy Dowle[1*]

[1] Department of Anatomy, University of Otago, Dunedin 9016, New Zealand

[2] Department of Zoology, University of Otago, Dunedin 9016, New Zealand

[*] Corresponding authors

Keywords: *Forficula auricularia*, Hybrid genome assembly, Repeatome, Genome annotation

Supplementary Table 1. Nanopore sequencing output

Number of Reads	Total Bases	Median Read Length	N50 Length	Median Read Quality
3,021,511.00	10,698,430,965.00	897.00	11,986.00	13.34

Supplementary Table 2. Assembly statistics after each round of processing

Assembly steps	Assembly length	No. of scaffolds	N50	L50	Ns per 100 kbp	Busco % (Quast)	
						Complete	Partial
Supernova	1,145,470,221	145,055	30,358	7,500	3,677.89	64.69	9.24
Flye	1,118,374,848	18,766	180,737	1,832	0.35	82.18	9.24
Quickmerge	1,138,492,550	16,670	222,450	1,435	1,028.63	84.16	5.94
Purgehaplotigs	1,092,680,447	11,914	233,917	1,342	1,053.46	84.49	5.94
Rails & Cobbler	1,094,435,828	10,883	251,120	1,250	851.82	84.82	5.61
Lrscaff	1,213,856,231	8,227	329,347	1,101	2,060.89	84.49	4.95
LrGapcloser	1,213,833,977	8,227	329,347	1,101	488.12	86.8	3.96
Ragtag	1,213,843,077	8,136	330,338	1,097	488.84	87.79	3.63
Arbit	1,211,594,836	6,512	529,318	681	500.51	87.13	3.63
Arks & Links	1,211,713,036	5,330	740,184	473	510.23	-	-
Rascaf	1,220,673,574	5,170	824,726	410	1,241.57	87.13	3.96

Purgehaplotigs	1,134,953,563	4,307	887,017	363	864.02	85.81	4.62
Ragtag	1,135,017,063	3,672	1,234,415	225	869.52	86.8	4.29
Blobtools	1,062,122,003	1,881	1,349,000	199	837.87	83.17	4.62
Ragtag	1,062,218,203	919	12,548,662	20	846.85	83.17	4.62
Pilon	1,062,210,345	919	12,548,649	20	846.85	87.13	2.97

(Note: Missing values are because of no data produced by Quast)

Supplementary Table 3. Functional annotation statistics from different databases

Database	No. of terms linked to mRNA	No. of mRNA with term	No. of gene with term
InterPro	17,976	12,904	7,666
Gene ontology term	15,088	7,669	4,560
Pfam	18,130	12,904	7,666

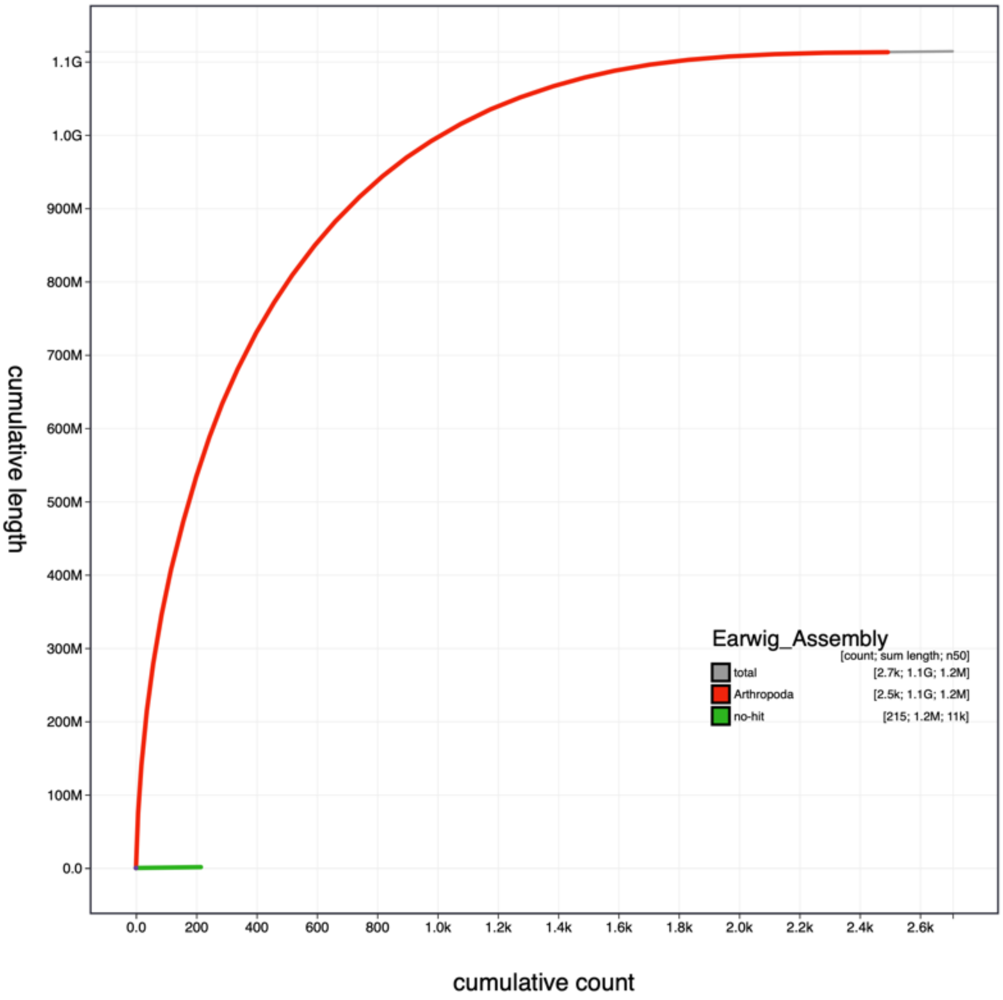

Supplementary Figure 1. Blast results after Blobtools2 filtering of the assembly. The red line shows the cumulative length of the scaffolds in assembly with blast hits to Arthropod database where as green line shows that with no-hits.

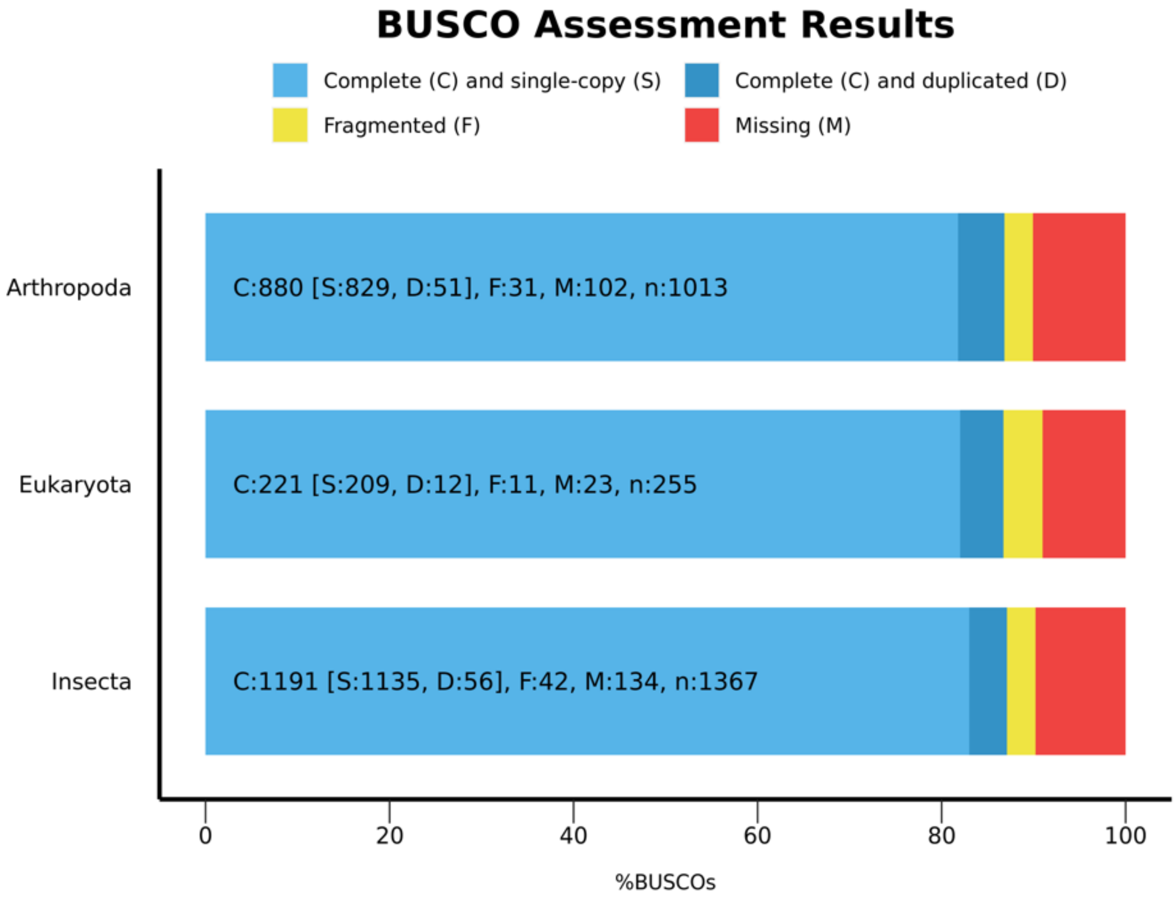

Supplementary Figure 2. The BUSCO report for the final hybrid assembly of the *F. auricularia* genome. BUSCO scores in percentage (x-axis) from Arthropoda, Eukaryota, and Insecta (Odb_10) databases (y-axis) are shown in the bar plot. The light blue portion of the bar represents complete and single-copy orthologs, dark blue represents complete and duplicated orthologs, yellow represents fragmented BUSCO genes and red represents missing BUSCO genes.

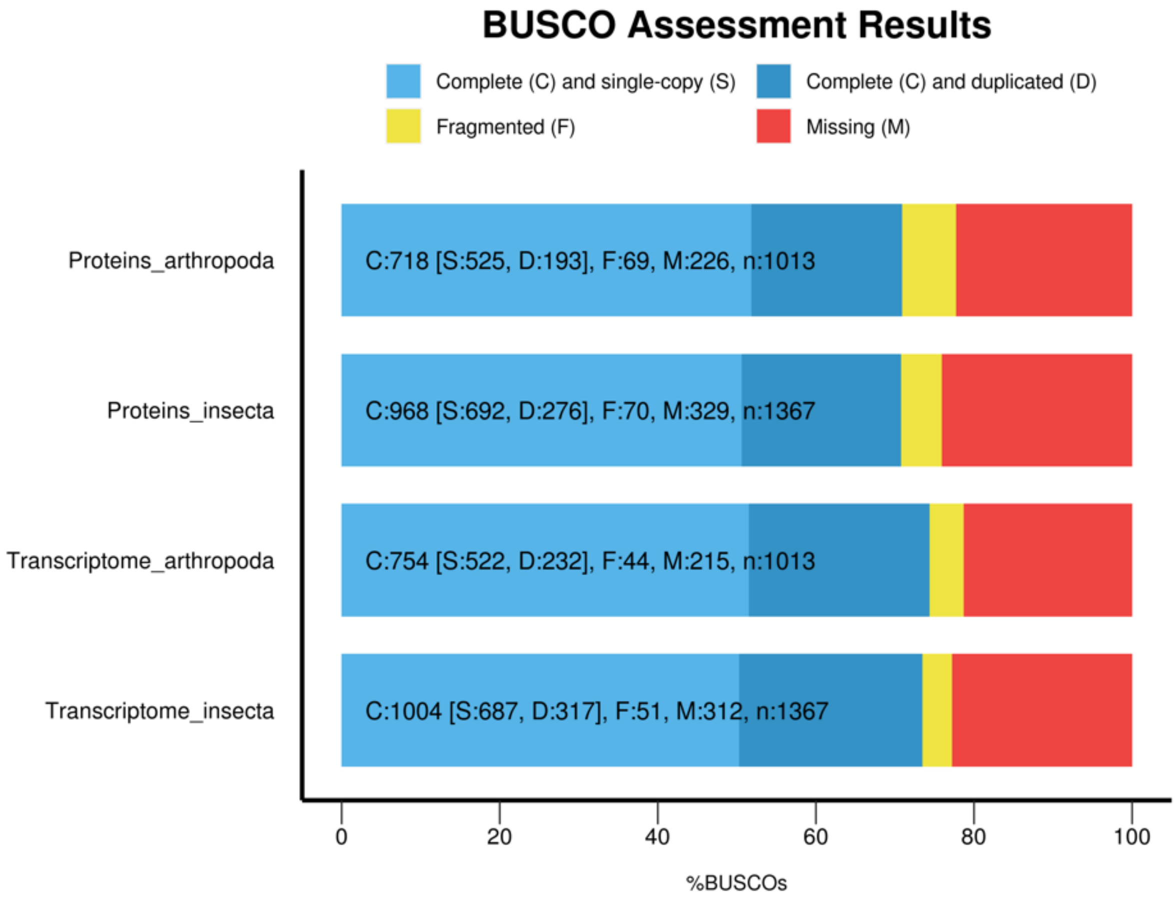

Supplementary Figure 3. Annotation completeness through BUSCO database. The plot shows the BUSCO percentage (x-axis) for the annotated proteins and transcriptomes using arthropoda_0db10 and insecta_odb10 database as indicated in the y-axis.

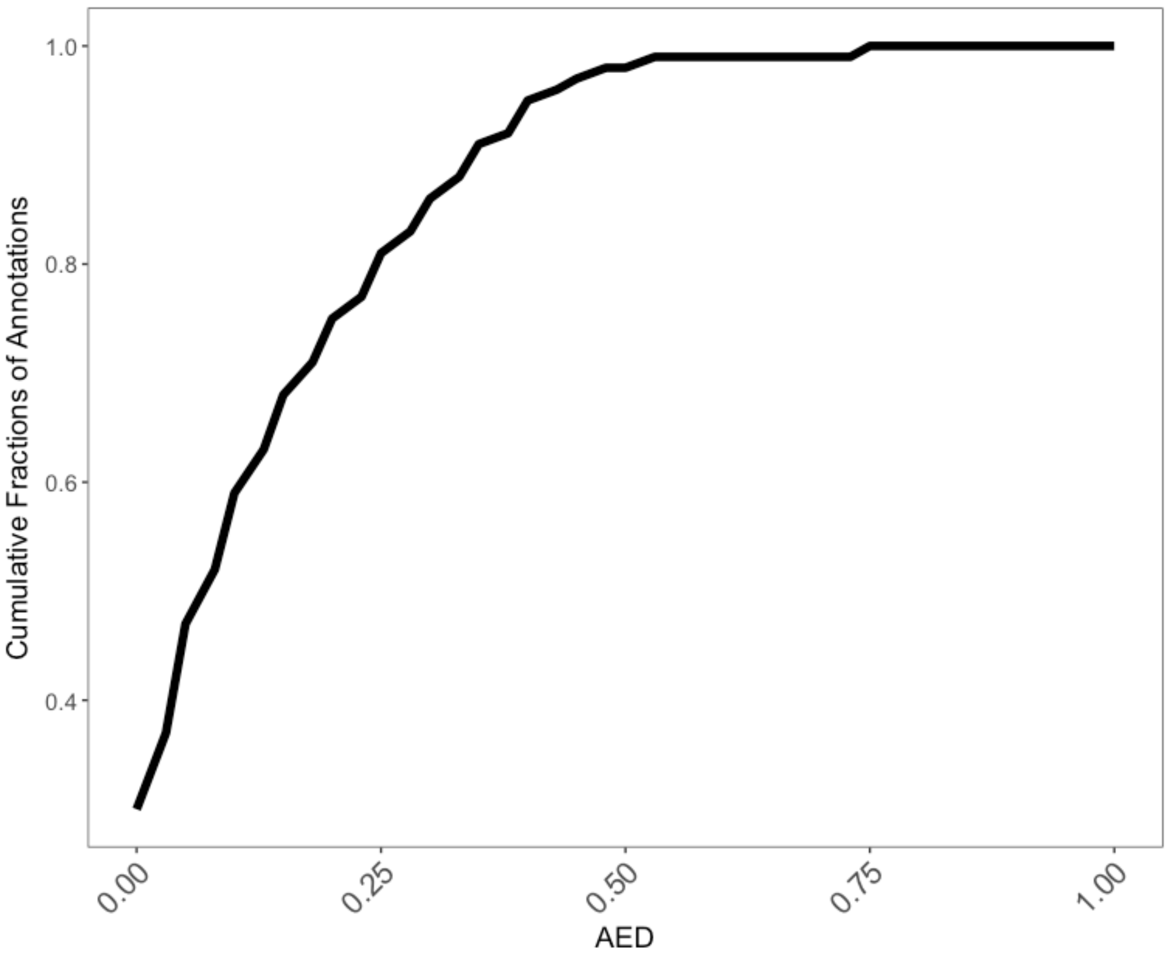

Supplementary Figure 4. Annotation quality with AED scores. Y-axis shows the cumulative fractions of annotations and x-axis their AED scores.
